# A New Laccase of Lac 2 from the White Rot Fungus *Cerrena unicolor* 6884 and Lac 2-Mediated Degradation of Aflatoxin B_1_

**DOI:** 10.3390/toxins12080476

**Published:** 2020-07-27

**Authors:** Zhimin Zhou, Renkuan Li, Tzi Bun Ng, Yunyun Lai, Jie Yang, Xiuyun Ye

**Affiliations:** 1 College of Chemical Engineering, Fuzhou University, Fuzhou 350116, China; n180410016@fzu.edu.cn; 2 The Key Laboratory of Marine Enzyme Engineering of Fujian Province, Fuzhou University, Fuzhou 350116, China; rkuanlee@fzu.edu.cn (R.L.); n180820012@fzu.edu.cn (Y.L.); T09136@fzu.edu.cn (J.Y.); 3 National Engineering Laboratory for High-efficient Enzyme Expression, Fuzhou 350116, China; 4 School of Biomedical Sciences, Faculty of Medicine, The Chinese University of Hong Kong, Shatin, New Territories, Hong Kong 999077, China; tzibunng@cuhk.edu.hk

**Keywords:** laccase, *Cerrena unicolor*, aflatoxin B_1_, biodegradation, detoxification, product, transcriptome

## Abstract

Aflatoxin B_1_ (AFB_1_) is a known toxic human carcinogen and can be detoxified by laccases, which are multicopper oxidases that convert several environmental pollutants and toxins. In this study, a new laccase that could catalyze AFB_1_ degradation was purified and identified from the white-rot fungus *Cerrena unicolor* 6884. The laccase was purified using (NH_4_)_2_SO_4_ precipitation and anion exchange chromatography, and then identified as Lac 2 through zymogram and UHPLC-MS/MS based on the Illumina transcriptome analysis of *C. unicolor* 6884. Six putative laccase protein sequences were obtained via functional annotation. The *lac 2* cDNA encoding a full-length protein of 512 amino acids was cloned and sequenced to expand the fungus laccase gene library for AFB_1_ detoxification. AFB_1_ degradation by Lac 2 was conducted in vitro at pH 7.0 and 45 °C for 24 h. The half-life of AFB_1_ degradation catalyzed by Lac 2 was 5.16 h. Acetosyringone (AS), Syrinagaldehyde (SA) and [2,2′ -azino-bis-(3-ethylbenzothiazoline-6-sulfonic acid)] (ABTS) at 1 mM concentration seemed to be similar mediators for strongly enhancing AFB_1_ degradation by Lac 2. The product of AFB_1_ degradation catalyzed by Lac 2 was traced and identified to be Aflatoxin Q_1_ (AFQ_1_) based on mass spectrometry data. These findings are promising for a possible application of Lac 2 as a new aflatoxin oxidase in degrading AFB_1_ present in food and feeds.

## 1. Introduction

**Figure 1:**
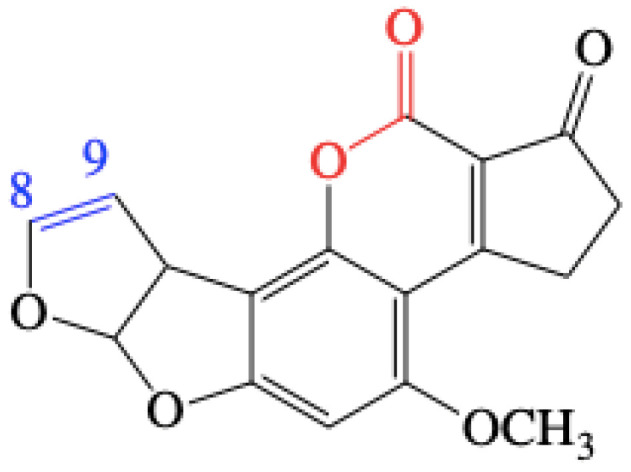
Chemical structure of aflatoxin B_1_ (AFB_1_). The C_8_-C_9_ double bond is highlighted in blue, while the lactone bond is indicated in red.

Aflatoxins (AFs), which are commonly produced by *Aspergillus* fungi such as *Aspergillus flavus* and *Aspergillus parasiticus,* are toxoids leading to fluorescence, human carcinogenicity and mutagenicity. The toxicity of AFs is related to the C_8_-C_9_ double bond of the difuran ring and the lactone ring within the coumarin ring [1,2]. Based on these data, AFs can be structurally classified into two main groups: the cyclopentenone group (e.g., aflatoxin B_1_ (AFB_1_, Figure 1), aflatoxin M_1_ (AFM_1_), aflatoxin Q_1_ (AFQ_1_), and aflatoxicol), and the cyclo-lactone group (e.g., aflatoxin G_1_ (AFG_1_) and its derivatives); AFB_1_, among the various aflatoxins, is commonly considered to be the most carcinogenic, inhibiting the synthesis of DNA, RNA and proteins [3]. AFB_1_, a difuranocoumarin derivative, is not only a known human carcinogen, but it can also affect a wide range of commodities [4,5]. AFB_1_ and its metabolites are mainly distributed in corn, peanuts, rice, wheat, oil by-products, dairy products and condiments [6,7]. It is estimated that over 5 billion people worldwide are at risk of chronic exposure to AFs in food [4].

Traditional methods of degradation of aflatoxin B_1_, including physical [8], chemical [9], and microbial approaches [10], suffer from the drawbacks of being time consuming, the loss of nutrients, lowering of quality, increased cost, and formation of toxic residues or derivatives [11,12]. Enzymatic degradation of AFB_1_ is effective and environmentally friendly, especially in food and feed industries [13]. Enzymes reportedly employed for AFB_1_ degradation comprise mainly oxidoreductase, including aflatoxin oxidase enzyme (AFO) [14], peroxidase [15], F_420_H_2_-dependent reductases (FDR) [16], Mn peroxidase (MnP) [11,17], myxobacteria aflatoxin degrading enzyme (MADE) [18], bacillus aflatoxin-degrading enzyme (BADE) [19] and laccases [20,21,22]. The mechanisms of AFB_1_ degradation for these enzymes have been elucidated since they have different targets on AFB_1_ molecules and different active sites: AFO [23,24] and MnP [11] act on the bifuran ring; FDR catalyzes reduction of α, β-unsaturated ester bond [16]; MADE acts on aromatic lactone and the methoxy group of the coumarin ring [18]. However, the mechanism of laccase-catalyzed AFB_1_ degradation has not been fully unraveled.

Laccases (EC 1.10.3.2) are copper-containing polyphenol oxidases which catalyze the oxidation of an array of aromatic substrates concomitantly with the reduction of molecular oxygen to water [25]. Laccases are produced by fungi for degrading lignin, humic compounds (phenols) and pollutant. However, few reports have focused on their effectiveness on toxins, like AFB_1_. Alberts et al. first found that the pure and recombinant laccases from *Trametes versicolor* could directly oxidize AFB_1_ with significant loss of mutagenicity, and its degradation products could not be detected after treatment using LCMS and HPLC [21]. In their hypothesis, the C_8_-C_9_ double bond in the furofuran ring might be responsible for the loss of the fluorescence and mutagenic properties of AFB_1_ with treatment of fungal laccases. Furthermore, the prooxidant properties and mutagenicity of the detoxification products obtained via laccase from *T. versicolor* have been studied by Hamed et al. [26]. Moreover, the interaction between AFs and laccases (*T. versicolor* [27] and *Saccharomyces cerevisiae* [28]), were studied by using 3D molecular techniques to gain an insight into the detailed mechanism of catalysis and decipher the reasons underlying the formation of degraded products.

Apart from *T. versicolor* laccases, Lac 2 from *Pleurotus pulmonarius* and Ery 4 from *Pleurotus eryngii* were also demonstrated by Loi et al. to degrade AFB_1_ via the mediation of natural phenolic compounds [22,29]. In their research, a laccase-mediator system (LMS) proved to enhance the degradation properties of laccases towards multiple mycotoxins. However, no degradation product of AFB_1_ catalyzed via LMS was identified. They hypothesized that the deeper modification of the coumarin-like core of the AFB_1_ would be responsible for the fluorescence quenching [22], which was different from the findings of Alberts et al. [21]. In addition, Wang et al. investigated whether CotA laccase from *Bacillus subtilis* (*Bs*CotA) was capable of detoxifying AFB_1_ and zearalenone (ZEN) using structurally defined chemicals and complex natural mediators, and the degraded products of AFB_1_ using the LMS were detoxified [20]. In more recent studies, Guo et al. found that recombinant CotA laccase from *Bacillus licheniformis* transformed AFB_1_ to AFQ_1_ and epi-AFQ_1_, which did not suppress cell viability or induce apoptosis [30]. As summarized above, both molecular docking and product analysis were employed to ascertain the mechanism of AFB_1_ degradation. Nevertheless, neither the mechanism of fungal laccase-mediated AFB_1_ degradation, nor the detoxification products, have been fully elucidated. Therefore, the tasks of screening new fungal laccases and possible degradation products still pose great challenges and will open up new perspectives at molecular and product levels to clarify the mechanism.

*Cerrena unicolor*, known as a white-rot fungus of the *Polyporaceae* family, was previously reported to be an excellent producer of fungal laccase with an ability to degrade environmental pollutants [31]. Many studies have been undertaken on laccases from *C. unicolor*, including purification, characterization, cloning, heterologous expression, transcriptomic analysis and application [32,33,34,35,36,37,38,39]. Since laccases from *T. versicolor* and *C. unicolor* always share similar enzymatic properties [21], the study of AFB_1_ degradation by *C. unicolor* laccase would be a practical project based on those mentioned above. Our hypothesis is that *C. unicolor* laccase would be a workable and efficient candidate for AFB_1_ detoxification. In this study, we purified and identified a new laccase from *C. unicolor* and investigated its capability for AFB_1_ degradation. Our results showed that laccase from *C. unicolor* was a valuable enzyme in AFB_1_ degradation. Moreover, efforts were undertaken to identity the laccase-AFB_1_ degraded products to hypothesize on the active sites.

## 2. Results

### 2.1. Species Identification

A fragment of the Internal Transcribed Spacer (ITS) gene of the target fungal strain 6884 was amplified by PCR and sequenced, and 6884 was classified based on its sequence. A phylogeny tree (Figure S1) was constructed, indicating that 6884 was closest to *Cerrena unicolor* (GenBank accession No. FN907915.1), *Cerrena unicolor* strain 042 (EU661887.1), and three more *Cerrena unicolor* strains (MK937761.1, MH855029.1, MH979248.1), sharing 100% sequence identity. The strain was named *Cerrena unicolor* 6884.

### 2.2. Preparation and Purification

**Figure 2:**
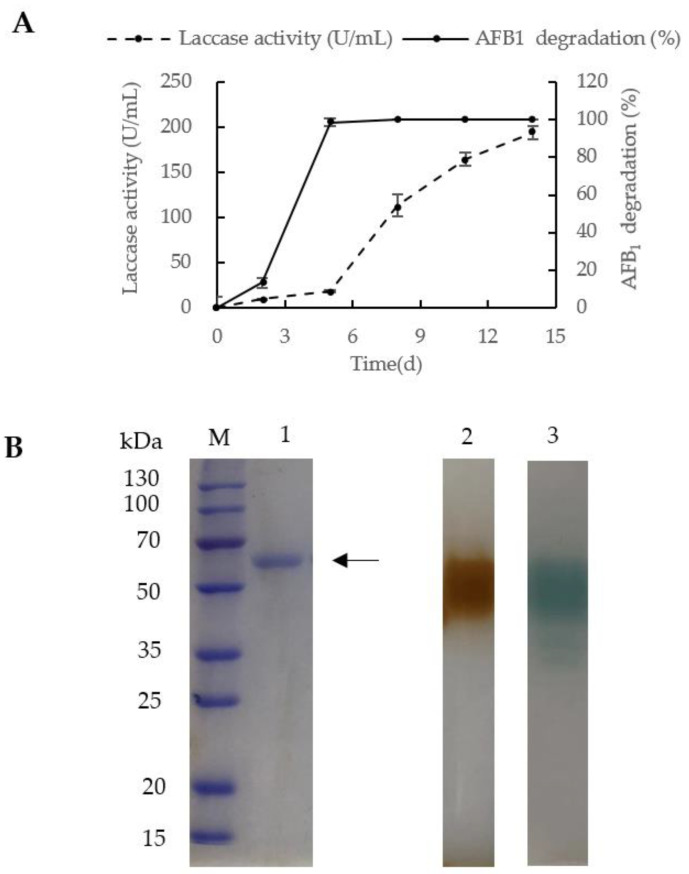
Preparation and zymogram of purified laccase from *C. unicolor* 6884. (**A**) Time course of laccase activity and AFB_1_ degradation mediated by the laccase. (**B**) Zymographic analysis of the purified laccase. Lane M: protein molecular weight marker by Takara; lane 1: purified laccase in SDS-PAGE; lane 2: purified laccase stained with guaiacol after electrophoresis; lane 3: purified laccase stained with 2,2′ -azino-bis-(3-ethylbenzothiazoline-6-sulfonic acid) (ABTS) after electrophoresis. The samples applied to lanes 2 and 3 had not been heated before loading. The arrow indicates the purified laccase. In A, the values represent means ± standard errors (*n* = 3).

After fermentation of *C. unicolor* 6884 for 14 days, laccase activity attained a maximum yield of 194 U/mL in the shake flask at 30 °C. On the fifth day of fermentation, *C. unicolor* 6884 produced laccase with an activity of 18.03 U/mL, with which, AFB_1_ at the concentration of 5 µg/mL could almost be all degraded (98.56%, Figure 2A). Meanwhile, 13.25% of AFB_1_ was degraded with laccase of fermentation broth possessing an activity of 9.54 U/mL on the second day. Considering the existence of other oxidases (e.g., peroxidase and MnP, both of which have been shown to degrade AFB_1_ effectively [11,15,17]) besides laccase in the culture broth, the AFB_1_ degradation of the culture broth was significantly improved even though the laccase activity was still in its lag phase on the fifth day. Thus, it is difficult to prove a correlation between the laccase activity and AFB_1_ degradation with the culture broth. However, the purified laccase activity and AFB_1_ degradation were positively correlated as shown in Figure S2.

After 14 days of fermentation, laccase was purified from the fermentation supernatant by employing a protocol involving ammonium sulfate precipitation and ion exchange chromatography (DEAE) (Table 1). The specific activity of laccase was 68.07 U/mg in the fermentation medium. After ion exchange chromatography, the specific activity was increased to 1643.70 U/mg, and the purification fold was 24.15. A purified protein band with a molecular weight of approximately 60 kDa was detected in SDS-PAGE (Figure 2B, lane 1). Laccase activity was shown after native electrophoresis using guaiacol and 2,2′ -azino-bis-(3-ethylbenzothiazoline-6-sulfonic acid) (ABTS) as substrates in lanes 2 and 3, respectively (Figure 2B).

### 2.3. Laccase Identification

An mRNA-Seq approach was employed to gain insight into the laccase gene expression profile of *C. unicolor* 6884 during routine growth on PDA plates. A high-quality database containing a total of 39,419,310 clean reads were obtained with a Q 30 Bases Ratio 95.33%. The total number of reads was assembled to conduct the Transcript (numbers: 56591) and the Unigene (numbers: 13682, redundancy removal) of *C. unicolor* 6884. The whole sequences in Unigene were annotated against nine databases, including NR (NCBI non-redundant protein sequences, number of genes: 8732, percentage: 63.82%), NT (NCBI nucleotide sequences, 1410, 10.31%), PFAM (Protein family, 4103, 29.99), KOG (euKaryotic Ortholog Groups, 4267, 31.19%), Swiss-Prot (A manually annotated and reviewed protein sequence database, 5500, 40.2%), KEGG (Kyoto Encyclopedia of Genes and Genomes, 1849, 13.51%), CDD (Conserved Domain Database, 5060, 36.98%), TrEMBL (A supplement of Swiss-Prot, 8692, 63.53%) and GO (Gene Ontology, 6846, 50.05%), to find genes encoding putative laccases. There were 23 genes in the Unigene functionally annotated to be putative laccases, and only six different sequences were obtained after blasting and assembling, named *lac 1*-*lac 6* (Table S1).

Based on the Illumina transcriptome analysis of *C. unicolor* 6884, six laccase cDNAs covering the four copper-binding motifs of fungal laccases (L 1–L 4) (Table S1) were annotated for the function of coding laccase. Information about the laccases is presented in Table S2. A database (DB) containing all six laccase protein sequences was customized for Proteome Discover software (version 2.2, 2018, Thermo Fisher Scientific, Germering, Germany).

Table 2 summarizes the identification of peptides that were digestion products from the single protein band in SDS-PAGE (Figure 2B, lane 1) with the highest score. As a result, 13 unique peptides were identified and assigned to Lac 2 from *C. unicolor* 6884 with 48% coverage. After purification and identification, laccase from *C. unicolor* 6884 was designated as Lac 2. The deduced Lac 2 protein with 512 amino acids demonstrated the greatest resemblance (79% identity) to laccase 2 precursor from *Cerrena* sp. HYB07 (Table S2). The 1536-bp cDNA of *lac 2* was cloned and submitted to GenBank with the accession number MT232811.

### 2.4. AFB_1_ Degradation by Lac 2 from Cerrena Unicolor 6884

**Figure 3:**
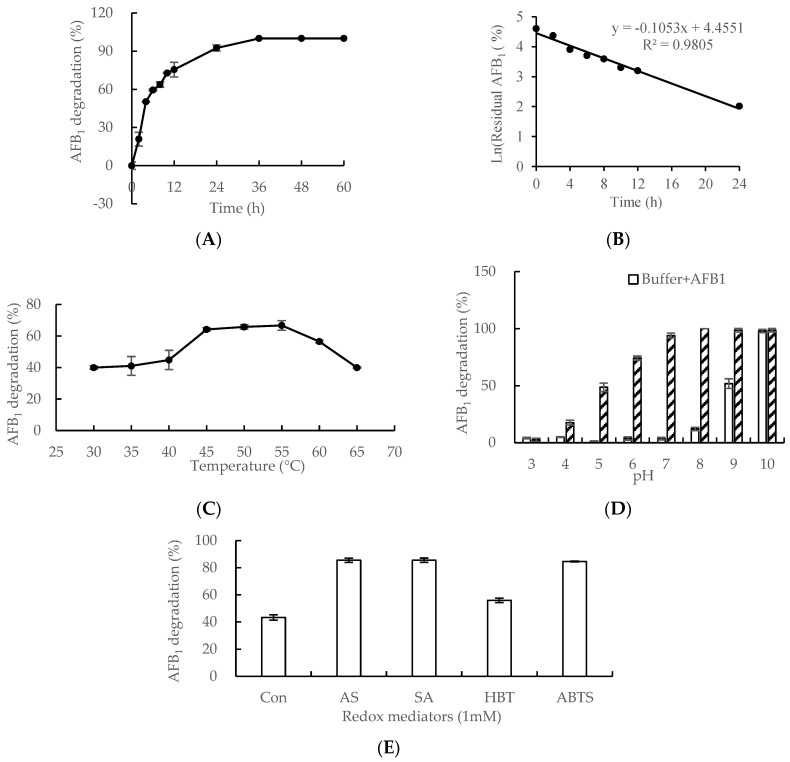
*Cerrena unicolor* 6884 Lac 2-mediated aflatoxin B_1_ (AFB_1_) degradation. (**A**) and (**B**) Time-course analysis of AFB_1_ degradation mediated by Lac 2. AFB_1_ (5 µg/mL) was incubated with Lac 2 (50 U/mL) in MilliQ water at 45 °C over a period of 60 h. Samples were periodically taken for HPLC. Effects of temperature (**C**), pH (**D**) and redox mediators (**E**) on AFB_1_ degradation by Lac 2. AFB_1_ (5 µg/mL) incubation with Lac 2 (50 U/mL) for 0 h were included as a treatment control. The residual AFB_1_ of all samples above was determined by a fluorescent detector (FLD) on HPLC (Agilent 1260 Infinity Ⅱ Series, Agilent Technologies, Waldbronn, Germany). The values represent means ± standard errors (*n* = 4).

Results of AFB_1_ degradation mediated by Lac 2 are shown in Figure 3. Fluorescence properties of the AFB_1_ molecule declined with the prolongation of the duration of incubation with Lac 2 as detected by a fluorescent detector (FLD) on HPLC (Agilent 1260 Infinity Ⅱ Series, Agilent Technologies, Waldbronn, Germany).

The time-course analysis of AFB_1_ degradation is shown in Figure 3A. The percentage of AFB_1_ degradation was 50% after an incubation of 4 h, increased steadily to 93% following an incubation of 24 h, and then gradually ascended to 100% at the end of an incubation period of 36 h. The half-life of AFB_1_ degradation catalyzed by Lac 2 was 5.16 h in this study (Figure 3B).

The optimal temperature for AFB_1_ degradation was investigated over a period of 8 h (Figure 3C). The percentage of AFB_1_ degradation was lower than 45% when the reaction temperature was below 45 °C. However, the AFB_1_ degradation rate increased rapidly from 45% to 65% when the temperature rose from 40 °C to 45 °C. The percentage of AFB_1_ degradation was maintained at about 65% in the temperature range of 45 °C to 55 °C. When the temperature was elevated to 60 °C and then to 65 °C, the percentage of AFB_1_ degradation fell to 55% and then to 41%. Therefore, the optimal temperature for AFB_1_ degradation mediated by laccase from *C. unicolor* 6884 lay between 45 °C and 55 °C, as shown in Figure 3C.

AFB_1_ degradation was analyzed over a range pH values (3.0–10.0) by incubation of AFB_1_ with Lac 2 for 24 h (Figure 3D). When the pH was lower than 7.0 (pH 3.0 to 7.0), AFB_1_ degradation could not proceed without Lac 2. The percentage of Lac 2-catalyzed AFB_1_ degradation was 94% when pH was 7.0. However, AFB_1_ was degradable in an alkaline buffer with a pH between 8.0 and 10.0. Thus, it is deduced that neutral pH was the optimal condition for AFB_1_ degradation by Lac 2 from *C. unicolor* 6884 (Figure 3D). The kinetic parameters, *K*_m_, *K*_cat_ and V_max_, of the Lac 2 towards AFB_1_ were 14.46 μM, 0.08 s^−1^and 1.30 μg min^−1^ mg^−1^, respectively.

Furthermore, different LMSs were studied for AFB_1_ degradation by Lac 2 over a period of 3 h (Figure 3E). AFB_1_ degradation by Lac 2 was almost doubled (85%, 85% and 83% vs. 44%, respectively) in the presence of 1 mM AS, SA and ABTS. Syringyl-type phenols (AS and SA) and ABTS enhanced AFB_1_ degradation mediated by Lac 2 to similar extents, while hydoxybenzotriazole (HBT) promoted AFB_1_ degradation mediated by Lac 2 less effectively.

### 2.5. Analysis of Products of AFB_1_ Degradation Catalyzed by Lac 2

**Figure 4:**
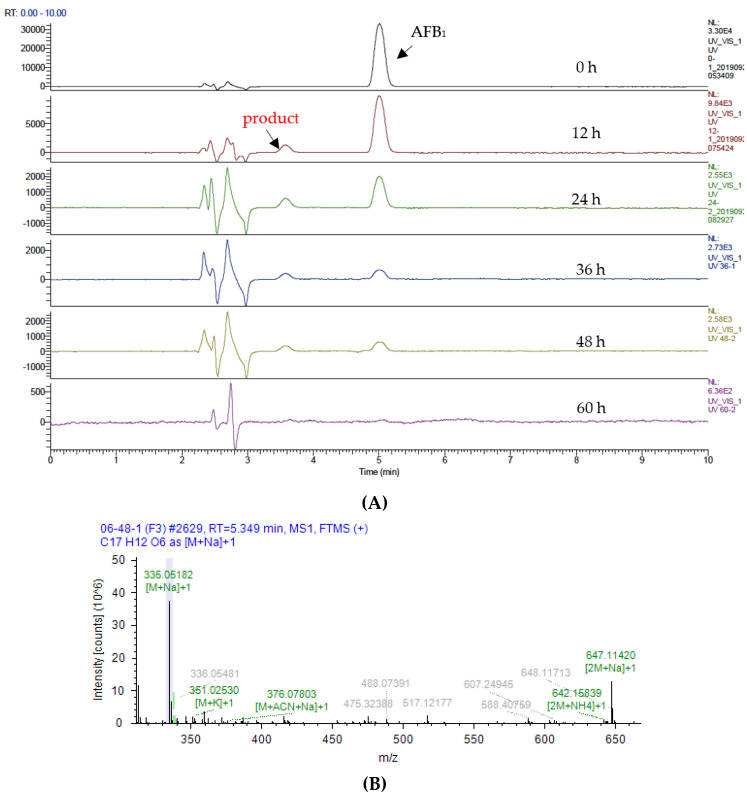

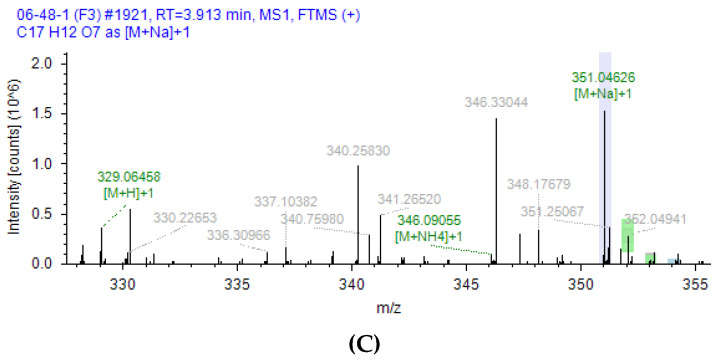
Mass spectral analysis of Lac 2-mediated aflatoxin B_1_ degradation. NSI-MS experiments were performed using a Full MS in the positive ion mode. (**A**) Lac 2-mediated AFB_1_ degradation process over a period of 60 h. Samples were periodically taken for UHPLC-MS/MS. AFB_1_ and the product at the retention time of 5.349 min and 3.913 min, respectively. (**B**) Mass spectrum of AFB_1_ (C_17_H_12_O_6_). (**C**) Mass spectrum of the product from Lac 2-mediated AFB_1_ degradation (C_17_H_12_O_7_).

As shown in Figure 4A, the products of AFB_1_ formed as a result of catalysis by Lac 2 were analyzed by UHPLC (UltiMate 3000 series, Thermo Fisher Scientific, Germering, Germany) with a diode array detector (DAD) and mass detector (Q Exactive mass spectrometer, Thermo Fisher Scientific, Germering, Germany). With the prolongation of incubation time, the percentage of residual AFB_1_ gradually declined to zero. In the process of AFB_1_ degradation, a new fraction emerged in the chromatogram at an intermediate stage at about 3.57 min and subsequently vanished. The new fraction in the chromatogram exhibited the molecular formula C_17_H_12_O_7_ ((M + H), and demonstrated ^+^ ion peaks at *m*/*z* 329.06458, (M + NH_4_) ^+^ ion peaks at *m*/*z* 346.09055, and (M + Na) ^+^ ion peaks at *m*/*z* 351.04626) by UHPLC-MS/MS using Compound Discover software (version 3.0, 2018, Thermo Fisher Scientific, Germering, Germany). The mass spectrum of AFB_1_ and product are shown in Figure 4B and Figure 4C, respectively.

## 3. Discussion

There were six genes encoding a hypothetical laccase in the genome of *C. unicolor* 6884 based on the results of Illumina transcriptome analysis. After purification and identification, the secreted laccase from *C. unicolor* 6884 was designated as Lac 2. The open reading frame (ORF) of *lac 2* gene encoded a full-length protein of 512 amino acids with an estimated molecular mass of about 60 kDa, which is in the range of molecular masses of most fungal laccases with regular three domains (50–70 kDa) [25]. Reports on laccases responsible for AFB_1_ degradation include the following: BsCotA from *B. subtilis* (Accession: AID81987.1) [20], laccase from *T. versicolor* (Accession: CAA77015.1 [21], PDB code: 1KYA [26,27]); Lac 2 from *P. pulmonarius* (Accession: AAX40733.1) [22]; and Ery4 from *P. eryngii* (Accession: CAO79915.1) [29]. Researchers have been trying to investigate the mechanism by employing both molecular techniques and direct analysis of degradation products. A homology model of AFB_1_ and laccase protein from *T. versicolor* has been analyzed by Dellafiora et al. [27]. The results showed that homology modeling was an effective analytical tool to assess the laccase–AFB_1_ interaction for explaining the mechanism of catalysis. It is necessary to acquire more laccase sequences to expand the library when studying the mechanism of laccase-catalyzed AFB_1_ degradation. In this study, we identified a new laccase from *C. unicolor* 6884, which demonstrated the ability to degrade AFB_1_.

After purification by (NH_4_)_2_SO_4_ precipitation and anion exchange chromatography, the specific activity of purified Lac 2 was 1643.7 U/mg with ABTS as substrate, which is similar to those reported from *Cerrena* sp. WR1 (1013.5 U/mg) [40], *C. unicolor* BBP6 (1215.9 U/mg) [39] and *Cerrena* sp. HYB07 (1952.4 U/mg) [31]. Results of SDS-PAGE and zymogram suggested that Lac 2 is a monomeric protein (Figure 2B) resembling most fungal laccases from *C. unicolor* GSM-019 (63.2 kDa) [41] and *Lepista nuda* (56 kDa) [42].

The plane-conjugated configuration of furofuran and cumarin rings is responsible for the AF fluorescence [43]. The fluorescence properties of the AFB_1_ molecule were determined with FLD on HPLC. Some laccases from white rot fungi are capable of degrading AFB_1_. Only 12.66% of AFB_1_ remained after catalysis by laccase from *T. versicolor* at pH 6.5 for 72 h [21]. *C. unicolor*, a strain of white rot fungi we studied, is a representative wood-degrading basidiomycete used to produce laccase for degradation of environmental pollutants due to its high enzymatic activity toward a broad range of substrates. In vitro, Lac 2 from *C. unicolor* 6884 catalyzed degradation of 92.5% AFB_1_ at 45 °C for 24 h, and then nearly 100% degradation after 36 h (Figure 3A).

AFB_1_ is thermostable for its conjugated structure formed by the C8-C9 double bond, and it would not be dilapidated even at a high temperature of 160 °C [44]. The enzyme-substrate reaction temperatures affect the enzyme activity and molecular motions. In our study, the optimal temperature range was between 45 °C and 55 °C (Figure 3C). The optimal temperature for laccase from *T. versicolor* was 35 °C [26]. In the case of CotA laccase from *B. subtilis*, the AFB_1_ degradation rate rose when temperature was raised from 20 to 50 °C, but the rate was not reduced when the temperature continued to increase to 80 °C [20]. The optimal temperature for laccase-mediated AFB_1_ degradation depends on the source and species of origin of the laccase.

Exposure of AFB_1_ to alkaline conditions caused the formation of nonfluorescent derivatives [45]. In an alkaline buffer, the lactone bond in AFB_1_ will be opened when attacked by nucleophiles, especially OHˉ, and then the detectability of AFB_1_ toxicity will be shielded, but the nonfluorescent compounds formed are strongly toxic based on results of the chick embryo’s test [12,46]. Our study showed similar results that buffers at pH 8.0, 9.0 and 10.0 were responsible for AFB_1_ degradation without laccase (Figure 3D). The extent of AFB_1_ degradation catalyzed by Lac 2 at pH 7.0 would be much greater than that in acidic buffers, which was similar to findings on CotA laccase from *B. subtilis* [20], but different from *T. versicolor* laccase with an optimal pH of 4.5. Furthermore, ABTS was studied as the substrate for the stability of Lac 2. After incubating Lac 2 at pH 6.0–9.0 and 25 °C for 24 h or 48 h, over 75% or 65% of the original enzyme activity was retained, respectively (Figure S3), indicating that Lac 2 was relatively more stable at pH of 6.0 or above. Therefore, the neutral condition was suitable for AFB_1_ degradation catalyzed by Lac 2. Considering the stability of Lac 2 and the better effect of Lac 2 on AFB_1_ degradation at higher pHs, the application of AFB_1_ degradation in the intestinal lumen of humans and non-ruminants presents an opportunity to investigate the potential applicability of feed enzymes in animal intestinal tracts. Therefore, in subsequent studies, Lac 2 will be immobilized to improve its resistance for further application.

LMS was efficient in improving degradation, as with purified laccases from the other white rot fungi. In this study, Lac 2 produced by *C. unicolor* 6884 degraded AFB_1_ (Figure 3E), which was similar to purified laccase from *T. versicolor* [21] and Lac 2 from *P. pulmonarius* [22]. As reported, Ery4-LMSs, 1 mM AS, SA and ABTS were able to double the degradation percentage compared to Lac 2 alone (85%, 85% and 83% vs. 44%, respectively), while HBT could improve degradation slightly. It took 13.86 h for the rate of AFB_1_ degradation catalyzed by Lac 2 to exceed 80% (Figure 3B), but only 3 h by addition of 1 mM AS, SA or ABTS in the reaction mixture (Figure 3E). Mediators efficiently improved AFB_1_ degradation. Considering the reaction modes, the AS, SA (natural 2,6-dimethoxy-substituted phenols) and HBT (artificial compounds) share the Hydrogen Atom Transfer (HAT) mechanism, while ABTS (artificial compounds) follows the Electron Transfer (ET) mechanism [47]. Therefore, the potential use of Lac 2 in treatment of raw materials could be advantageous due to its redox mediators and LMS high efficiency.

Few reports are available on the catalytic mechanism deployed by laccase in degradation of AFB_1_ and the degradation products. Several chemical reactions including epoxidation, hydroxylation, dehydrogenation and reduction, etc., [22], have been speculated as the potential mechanism of AFB_1_ degradation, but most are merely hypotheses and have not been corroborated. It is necessary to develop methods for AFB_1_ degradation debris tracking. A recent study on rCotA laccase from *B. licheniformis* revealed that AFB_1_ could be oxidized to AFQ_1_ and epi-AFQ_1_ [30]. However, studies on products derived from AFB_1_ formed by the catalytic action of a fungal laccase have not been described. We attempted to investigate the products of AFB_1_ degradation catalyzed by Lac 2 from *C. unicolor* 6884 in order to speculate a mechanism of action of fungal laccase.

In our study, a new conspicuous fraction eluted at about 3.57 min in the UHPLC chromatogram (Figure 4A) emerged at an intermediate stage but eventually became undetectable. The same results were observed when AFG_1_ was degraded with the catalytic assistance of Lac 2 (data not shown). Thus, it can be concluded that the cyclopentenone structure of AFB_1_ and the cyclo-lactone structure of AFG_1_ were not the target sites for Lac 2. The new fraction had the molecular formula C_17_H_12_O_7_, which showed the molecular ion peak at *m/z* 329.06458 ([M + H] ^+^), 346.09055 ([M + NH_4_] ^+^), and 351.04626 ([M + Na] ^+^). Compared to AFB_1_ (C_17_H_12_O_6_), this formula shows addition of only one more oxygen, and it is considered to be an AFB_1_ metabolite. According to the results of UHPLC-MS/MS in Figure 4A and Figure S4, the concentration of AFB_1_ gradually decreased to zero after incubation for 60 h. In the whole process, the AFB_1_ metabolite increased in the first 4 h, and then declined as AFB_1_ appeared. Therefore, part of AFB_1_ would be converted to the metabolite, which would be slowly degraded, such as that of AFB_1_ via Lac 2 during the following incubation.

To date, AFB_1_, AFB_2_, AFM_1_, AFG_1_ and AFG_2_, can be detoxified with laccases [22,28]. As we know, AFB_1_ can undergo in vivo hydroxylation to its derivatives, AFM_1_, AFQ_1_, or aflatoxicol [1]. In our hypothesis, the molecular formula of C_17_H_12_O_7_ for the AFB_1_ metabolite is an AFB_1_-like structure, probably AFM_1_, AFQ_1_, epi-AFQ_1_, AFG_1_, AFG_2_ or 8,9-AFB_1_ epoxide, for these aflatoxins sharing the same *m/z* and molecular formula. According to Loi et al. [48], comparing the MS/MS spectrum of the unknown AFB_1_ metabolite to equivalent spectra of all known AFs can give a clue about the identity of this metabolite. AFQ_1_ is a known AFB_1_ metabolite; however, it is not commercially available. The MS/MS spectra of AFQ_1_ was cited as a reference by Loi et al. [48], in which Dr. De Boevre generously provided the MS/MS spectra of AFQ_1_.

A comparison of the MS/MS spectra of AFB_1_ metabolite obtained in this study and equivalent spectra of AFQ_1_ [48] revealed a matching of most diagnostic ions, e.g., m/z 174.99153, 176.98946, 206.05647, 283.05804, 311.05396 and 330.06827 (Figure S5). Based on these findings, the unknown AFB_1_ metabolite hydroxylated by Lac 2 was extremely likely to be AFQ_1_.

## 4. Conclusions

In this study, the next-generation sequencing technology (NGS, Illumina transcriptome analysis) was deployed, and a high-quality Unigene was conducted and annotated against nine databases to a customized database for direct identification of a new laccase and cloning of its cDNA sequence. Six laccase cDNAs covering the four copper-binding motifs of fungal laccases were annotated for the function of coding laccase. A new Lac 2 that catalyzed AFB_1_ degradation was purified and identified from the white-rot fungus *Cerrena unicolor* 6884. The cDNA of *lac 2* was cloned and submitted to GenBank. The extent of AFB_1_ degradation achieved at pH 7.0 and 45 °C and an incubation duration of 24 h with laccase as the enzyme was 94%. The stability of Lac 2, and the more extensive degradation of AFB_1_ at higher pH values suggest the potential applicability of feed enzymes in the animal intestinal tract. The freely additional of redox mediators and LMS high efficiency enhanced the potential applicability of Lac 2 in the treatment of raw materials. Additional studies on the immobilization of Lac 2 and Lac 2-mediated AFB_1_ degradation in the animal intestinal tract and raw food material processing are required.

The Lac 2-mediated AFB_1_ degradation product is surmised to be an AFB_1_-like structure through UHPLC-MS/MS. AFB_1_ may be converted to AFB_1_ metabolite partially with the catalytic assistance of Lac 2, and all will then be transformed to degradation products with chemical properties that vastly differ from the AFs. Comparing the MS/MS spectra of AFB_1_ metabolite with equivalent spectra of AFQ_1_ led to the conclusion that the AFB_1_ metabolite was AFQ_1_. To our knowledge, this identification of the product represents the first characterization of the role of fungal laccase in AFB_1_ detoxification. The complete unraveling of the mechanism of AFB_1_ degradation induced by a laccase from white-rot fungus necessitates further investigations. Besides analysis of the degradation products, molecular technology will also be a tool for elucidating the reasons underlying the formation of degraded products.

## 5. Materials and Methods

### 5.1. Organism, Chemicals and Other Materials

The *Cerrena unicolor* 6884 strain was purchased from China Forestry Culture Collection Center (CFCC). pMD-18 T vector Kit was purchased from TAKARA (Dalian, Liaoning, China). E. coli Top10 and RevertAid First Strand cDNA Synthesis Kit was purchased from Thermo Scientific (Vilnius, Lithuania). Total RNA Extractor (Trizol) and BCA Protein Assay Kit were purchased from Sangon (Shanghai, China). Illumina transcriptome analysis and primer synthesis were performed by Sangon (Shanghai, China) and sequencing reactions were performed by Invitrogen (Guangzhou, Guangdong, China).

Chemicals for gel electrophoresis were supplied by Solarbio (Beijing, China) and TaKaRa (Dalian, Liaoning, China). Trypsin (proteomic grade), trizol, and SA (Syrinagaldehyde) were purchased from Thermo Fisher Scientific (Waltham, MA, USA). AFB_1_ standards (purify ≥ 98%) and ABTS (2-azino-di-[3-ethylbenzo-thiazolin-sulphonate]) were purchased from Sigma-Aldrich (St. Louis, MO, USA). AS (Acetosyringone) was purchased from Solarbio (Beijing, China). HBT (N Hydroxy benzorizole) was purchased from Aladdin (Shanghai, China). Acetonitrile (ACN), Methanol, formic acid (FA), and trifluoroacetic acid (TFA) used in mass spectrometry of HPLC grade were purchased from Merck (Darmstadt, Germany). Regenerable cellulose syringe filters, 0.22 μm (size 13 mm), were obtained from Jinteng (Tianjin, China). Other chemicals used were of analytical grade from Sinopharm (Shanghai, China).

### 5.2. Phylogenetic Analysis

Phylogeny of the strain was identified by using ITS sequencing. Genomic DNA was extracted with a DNA Quick Plant System (TIANGEN, Beijing, China), and universal primers ITS 1 and ITS 4 were used for amplification of ITS. The PCR product was sequenced. The ITS sequence has been submitted to GenBank with the accession number MT712199. The phylogenetic analysis (with 1000 bootstraps) was performed with MEGA version 7.0 by the neighbor-joining method. Other fungal ITS sequences used in this study were from GenBank.

### 5.3. Illumina Transcriptome Analysis of Cerrena Unicolor 6884

*C. unicolor* 6884 was routinely grown on PDA plates at 30 °C for 4 days. The extraction of total RNA and the Illumina transcriptome analysis were both performed by Sangon. An mRNA-Seq approach was employed to gain insight into the laccase gene expression profile of *C. unicolor* 6884 during routine growth on PDA plates. The total number of reads was assembled with Trinity (Version 2.4.0) to conduct the Transcript and Unigene, and the Unigene of *C. unicolor* 6884 was submitted to NCBI with the SRA accession PRJNA644218. The whole sequences in Unigene were blasted against nine databases, including NR, NT, PFAM, KOG, Swiss-Prot, KEGG, CDD, TrEMBL and GO, to find genes encoding putative laccases. These putative laccase genes have been submitted to GeneBank with accession numbers MT720691, MT720692, MT720693, MT720694, MT720695 and MT720696, respectively.

### 5.4. Production of Laccase

The production of laccase was carried out as previously described in [38]. Briefly, *C. unicolor* 6884 was grown in 50 mL medium (2% *v/v* glycerol, 1.5% *w/v* peptone, 6 g KH_2_PO_4_, 4.14 g MgSO_4_·7H_2_O, 0.3 g CaCl_2_, 0.18 g NaCl, 0.0625 g CuSO_4_·5H_2_O, 0.018 g ZnSO_4_·7H_2_O, 0.015 g VB_1_, 1000 mL H_2_O) in 250 mL Erlenmeyer flasks at 30 °C with shaking at 200 rpm. Samples were collected at regular time intervals (0, 2, 5, 8, 11 and 14 d, respectively) for enzyme activity assays and AFB_1_ degradation in vitro described below. The reaction mixture of AFB_1_ degradation contained cultured broth and AFB_1_ (5 µg/mL) incubated at 45 °C over a period of 60 h. The samples were removed for HPLC analysis (Agilent 1260 Infinity Ⅱ Series, Agilent Technologies, Waldbronn, Germany) as detailed underneath. The values represent means ± standard errors (*n* = 3).

### 5.5. Enzyme Assay

Laccase activity was measured photometrically at 420 nm (Ɛ = 36,000 M^−1^·cm^−1^) according to the procedure described in [38]. The 2 mL reaction system contained 100 mM pH 3.0 sodium acetate solution (975 μL), 0.5 mM ABTS (1000 μL) and an appropriate amount of enzyme solution (25 μL). One unit was defined as the amount of enzyme which oxidized 1 μmol of substrate per min. All measurements were carried out in triplicate.

### 5.6. Purification of Laccase

After cultivation for 14 d, the fermentation culture was harvested, collected, centrifuged and fractionated by addition of 50% to 90% (NH_4_)_2_SO_4_. The precipitate was resuspended and desalted by dialysis against 20 mM Tris-HCl buffer (pH 8.5) and applied to a HiTrap DEAE column (15 cm × 5 cm, 5mL, GE Healthcare Bio-Science Corp, Piscataway, USA) on an ÄKTA^TM^ Purifier (GE Healthcare Bio-Science AB, Uppsala, Sweden). Adsorbed proteins were eluted with 0.2 M NaCl in 20 mM Tris-HCl buffer (pH 8.5) and ultra-filtered with a membrane module (nominal MW cut-off 10-kDa) at 4000× *g* for 20 min. Electrophoretic analyses were performed according to Yang [31] with a little modification. Briefly, fractions with laccase activity were examined by SDS-PAGE on 12% gels stained with Coomassie brilliant blue R-250 (Sigma, St. Louis, MO, USA) and zymography (stained with 0.04 mM guaiacol and 0.25 mM ABTS, respectively). Protein concentration was quantified by BCA Protein Assay Kit.

### 5.7. Identification of Laccase by UHPLC-MS/MS

The identification of laccase from *C. unicolor* 6884 by UHPLC-MS/MS was performed by cutting the laccase bands obtained by SDS-PAGE analysis. Protein digestion was accomplished according to the manufacturer’s instructions with slight modifications, with trypsin incubation overnight at 37 °C [22]. Digestion products were subsequently analyzed by UHPLC-MS/MS for peptide sequences on UltiMate 3000 RSLCnano/Q Exactive mass spectrometer (Thermo Fisher Scientific, Germering, Germany). NSI-MS experiments were performed using a Full MS/ddMS^2^ mass spectrometer system in the positive ion mode. Full MS was in the range 100–5000 *m*/*z*. Peptide separation was performed on an Acclaim PepMap^TM^ RSLC-C18 analytical column (75 μm × 15 cm, 2 μm, 100 Å, nanoViper, Thermo Fisher Scientific, USA). The injection volume was 5 μL. The following linear elution gradient was used for the analytical separation: solvent B remained at 2% for 3 min at the beginning of the elution, it was then varied from 2% to 6% in 3 min, from 6% to 28% in 45 min, from 28% to 34% in 4 min; then it was increased up to 95% in 2 min and this ratio was maintained constant for the following 13 min. The percentage of B suddenly decreased at 2% and kept stable for 10 min for column reconditioning. The two reserves used were: A = 2% ACN + 98% H_2_O + 0.04% FA and B = 80% ACN + 20% H_2_O + 0.08% FA; flow rate was set at 300 μL/min. The data were acquired and analyzed by Proteome Discover (version 2.2, 2017, Thermo Fisher Scientific, Germering, Germany).

### 5.8. Cloning of the Laccase cDNA from Cerrena Unicolor 6884

The strain *C. unicolor* 6884 was grown on PDA plates at 30 °C for 4 d. Total RNA was extracted with Total RNA Extractor (Trizol). The cDNA of *C. unicolor* 6884 was generated by RT-PCR from total RNA with RevertAid First Strand cDNA Synthesis Kit. *Lac 2* was amplified by PCR with the primer pair (*lac 2*-F: 5′-ATGGGATTGAACTCGGCT-3′; *lac 2*-R: 5′-TTAAATAGCAGTTCCTTTCTTAGGC-3′) using total cDNA of *C. unicolor* 6884 as the template. PCR products were inserted into the pMD18-T vector and transformed into *E. coli* top10 competent cells which were then cultured on an LB plate with 100 mg/mL ampicillin. Three positive clones of each fragment were sequenced. The cDNA sequence of *lac 2* studied in this paper has been submitted to GenBank with the accession number MT232811.

The sequences of *C. unicolor* 6884 laccases were analyzed online BLAST (http://blast.ncbi.nlm.nih.gov/Blast.cgi). SignalP-5.0 was used for theoretical signal peptide determination (http://www.cbs.dtu.dk/services/SignalP/).

### 5.9. In Vitro Degradation of AFB_1_ with Lac 2

For AFB_1_ degradation by Lac 2, a time-course analysis was carried out in the dark. The 200 μL reaction mixture contained 30 μg/mL Lac 2 (50 U/mL) and AFB_1_ (5 µg/mL) in MilliQ water at 45 °C over a period of 60 h. The samples were periodically (at 2 h, 4 h, 6 h, 8 h, 10 h, 12 h, 24 h, 36 h, 48 h, and 60 h) taken for HPLC analysis.

To investigate the effect of temperature on Lac 2-mediated AFB_1_ degradation, AFB_1_ (5 µg/mL) was individually incubated with Lac 2 (50 U/mL) in Na_2_HPO_4_-citric acid buffer (pH 7.0) at various temperatures ranging from 30 to 65 °C (30, 35, 40, 45, 50, 55, 60, and 65 °C) over a period of 8 h.

To investigate the effect of pH on AFB_1_ degradation by Lac 2, AFB_1_ (5 µg/mL) was individually incubated with or without Lac 2 (50 U/mL) for 24 h at 45 °C in buffers at different pH values, including Na_2_HPO_4_–citric acid buffers (pH 3.0–8.0), and 50 mM glycine-NaOH buffers (pH 9.0–10.0), respectively.

To determine the kinetic parameters (*K*_m_, *K*_cat_ and V_max_) for AFB_1_ degradation, the initial reaction rate was investigated by monitoring the removal of AFB_1_ (initial concentration from 1, 2, 3, 4, 5, 10, 20, and 25 μg/mL) at 10 min intervals up to 50 min at 45 °C and pH 7.0 with 30 μg/mL of Lac 2 (50 U/mL). The kinetic parameters were determined by nonlinear regression of Michaelis- Menten plots using the software GraphPad Prism 8.2.1 (San Diego, CA, USA). One unit of AFB_1_-degratation activity was defined as the quantity of laccase that detoxified 1 μg of AFB_1_ per minute.

To investigate the effect of LMS on AFB_1_ degradation, ABTS, AS, SA or HBT was independently tested as redox mediators at 1 mM with Lac 2 (50 U/mL) in Na_2_HPO_4_–citric acid buffer (pH 7.0) at 45 °C for 3 h, while controls did not have any mediator.

Incubation of AFB_1_ (5 µg/mL) with Lac 2 (50 U/mL) for 0 h was included as a treatment control. The residual AFB_1_ in all aforementioned samples was determined by FLD on HPLC (Agilent 1260 Infinity Ⅱ Series, Agilent Technologies, Waldbronn, Germany). The values represent means ± standard errors (*n* = 4).

### 5.10. AFB_1_ Assay

AFB_1_ was extracted from the samples with an equal volume of dichloromethane three times and evaporated under nitrogen gas at 50 °C. The samples were dissolved in an equal volume of methanol and filtered (0.22 μm, Jinteng, China).

HPLC analyses of the samples were performed on an Agilent 1260 Infinity Ⅱ Series (Agilent Technologies, Waldbronn, Germany) using a Poroshell 120 EC-C18 column (2.1×50 mm, 1.9 μm, Agilent Technologies, Waldbronn, Germany). The mobile phase for elution was composed of methanol, aceto-nitrile and water (1:1:2, *v*/*v*/*v*) at a flow rate of 0.1 mL/min for 5 min at 40 °C. The sample injection volume was 2 μL. AFB_1_ was measured by a FLD Detector which was set at the wavelengths of 365 nm (excitation) and 450 nm (emission). The retention time of AFB_1_ was 2.5 min.

The percentage of AFB1 degradation was calculated according to the following formula:

D (%) = (A_0_ − A)/A_0_ × 100, (1)

where D was the degradation efficiency (%), and A_0_ and A represented the peak area of AFB_1_ before and after degradation, respectively.

### 5.11. UPLC-MS/MS Analysis of AFB_1_ Degradation Products

The Lac 2-mediated AFB_1_ degradation products were detected by UHPLC-MS/MS. The degradation reaction was carried out in the dark at 45 °C for 0 h, 2 h, 4 h, 6 h, 8 h, 10 h, 12 h, 24 h, 48 h, and 60 h. The Lac 2-mediated AFB_1_ degradation products were analyzed by UHPLC (Thermo Scientific UltiMate 3000 System, Thermo Fisher Scientific, Germering, Germany) coupled with a Q Exactive mass spectrometer (Thermo Fisher Scientific, Germering, Germany). NSI-MS experiments were performed in the positive ionization mode. The mobile phase, which consisted of water, methanol and acetonitrile (1:1:2, *v*/*v*/*v*), was used for elution at a flow rate of 0.1 mL/min for 10 min at 40 °C. An Hypersil Gold C_18_ column (100 × 2.1 mm, 1.9 μm, Thermo Fisher Scientific, Germering, Germany) was used for separation at a flow rate of 0.1 mL/min for 10 min at 40 °C. The sample injection volume was 2 μL. AFB_1_ was measured by a DAD Detector at 365 nm. The retention time of AFB_1_ was 5.0 min. The AFB_1_ degradation products were identified and analyzed with the Compound Discover (version 3.0, 2018, Thermo Fisher Scientific, Germering, Germany).

## Figures and Tables

**Table 1:** Summary of laccase purification from culture filtrate of *Cerrena unicolor* 6884.

Purification Step	Total Activity (U)	Total Protein (mg)	Specific Activity (U/mg)	Purification Fold	Recovery (%)
Crude extract	44,641.67	655.86	68.07	1.00	100
NH_4_(SO_4_)_2_	33,653.33	163.49	205.84	3.02	75.39
DEAE cellulose	6592.00	4.01	1643.70	24.15	14.77

**Table 2:** Analysis of the excised band using UHPLC-MS/MS in combination with Protein Discovery.

Assigned Protein	Coverage [%]	Peptides	Sequence	Confidence	Theo. MH+ [Da]
Lac 2 from *Cerrena.* *unicolor* 6884	48	13	VVELVIPPLAVGGPHPFHLHGHNFWVVR	High	3123.71556
			TVGGPAQSPLNEADLRPLVPAPVPGNAVPGGADINHR		3653.91467
			SQTGPADAELAVISVEHNKR		2122.08872
			SQTGPADAELAVISVEHNK		1965.98761
			SAGSDEYNFDDAILRDVVSIGAGTDEVTIR		3185.52331
			SAGSDEYNFDDAILR		1672.74492
			NASVEEPK		873.43124
			NAAILR		657.40423
			MLTPTSIHWHGFFQK		1845.91049
			YSFVLNANQPDDNYWIR		2114.99303
			MLTPTSIHWHGFFQK		1829.91557
			GAFVVYDPNDPHK		1458.70120
			DVVSIGAGTDEVTIR		1531.79623
			DLYDVDDESTVITLADWYHVLAQTVVGAATPDSTLINGLGR		4404.18816

## Supplementary Materials

The following are available online at https://www.mdpi.com/2072-6651/12/8/476/s1, Table S1. Amino acid sequence alignment of the four conserved copper-binding sites of laccases from *Cerrena unicolor* 6884, Table S2. Information about laccases from *Cerrena unicolor* 6884, Figure S1. Phylogenetic relationships of *Cerrena unicolor* 6884 and related species based on ITS sequences. The numbers following the strains are accession numbers of ITS sequences. Bootstrap values at nodes are percentages of 1000 replicates. Scale bar indicates base substitutions/100 bases, Figure S2. The correclation between Lac 2 from *Cerrena unicolor* 6884 and aflatoxin B1 (AFB_1_) degradation. AFB_1_ (5 µg/mL) was individually incubated with Lac 2 ranging from 0 U/mL to 50 U/mL in MilliQ water at 45 °C over a period of 60 h. Incubation for 0 h were included as treatment controls. The residual AFB_1_ of all samples above was determined by FLD on HPLC (Agilent 1260 Infinity Ⅱ Series, Agilent Technologies, Waldbronn, Germany). The values represent means ± standard errors (n = 4), Figure S3. Effects of pH on stability of Lac 2. pH stability was studied by incubating the purified enzyme at different pH values in 20 mM Na2HPO4–citric acid buffers (pH 2.0–7.0), or 20 mM Na2HPO4–NaOH buffers (pH 9.0–10.0) at 25 °C for 24 h or 48 h, and the residual laccase activity was quantified at the optimum pH and temperature using ABTS as the substrate. The activity of the untreated enzyme was taken as 100%. The values represent means ± standard errors (n = 3), Figure S4. Biological degradation of aflatoxin B_1_ (AFB_1_) by laccase Lac 2 (50U/mL) produced by *Cerrena unicolor* at pH 7.0 and at 45 °C over a period of 0, 2, 4, 6, 8, 10, 12, 24, 36, 48, and 60 h. The residual AFB_1_ and intermediate product were determined by UHPLC (Thermo Scientific UltiMate 3000 System, Thermo Fisher Scientific, Germering, Germany). The values represent means ± standard errors (n = 3), Figure S5. MS/MS spectra of unknown AFB_1_ metabolite.

## Abbreviations

ABTS	[2,2′ -azino-bis-(3-ethylbenzothiazoline-6-sulfonic acid)]
ACN	Acetonitrile
AFB_1_	aflatoxin B_1_
AFG_1_	aflatoxin G_1_
AFM_1_	aflatoxin M_1_
AFQ_1_	aflatoxin Q_1_
AS	Acetosyringone
DAD	Diode array detector
DEAE	diethylamino ethyl
FLD	Fluorescent detector
HBT	Hydroxy benzotriazole
Lac 2	laccase 2
LMS	laccase mediator system
SA	Syrinagaldehyde
