# Supplementary material for: A New Laccase of Lac 2 from the White Rot Fungus Cerrena unicolor 6884 and Lac 2-Mediated Degradation of Aflatoxin B1
Source: Toxins (Basel). 2020 Jul 27;12(8):476. doi: 10.3390/toxins12080476 (PMC7472184; doi:10.3390/toxins12080476)
Supplement: Supplementary file 1 [file toxins-12-00476-s001.pdf]

Supplementary Materials: A New Laccase of Lac 2 from the White Rot Fungus *Cerrena unicolor* 6884 and Lac 2-Mediated Degradation of Aflatoxin B1

Zhimin Zhou, Renkuan Li, Tzi Bun Ng, Yunyun Lai, Jie Yang and Xiuyun Ye

Table S1. Amino acid sequence alignment of the four conserved copper-binding sites of laccases from *Cerrena unicolor* 6884.

Laccase	L 1	L 2	L 3	L 4
Lac 2	IHWHG	WYHSHLSTQ	HPFHLHGH	LHCHIDWH
Lac 4	IHWHG	WYHSHLSTQ	HPFHLHGH	LHCHIDWH
Lac 5	IHWHG	WYHSHLSTQ	HPFHLHGH	LHCHIDWH
Lac 6	IHWHG	WYHSHLSTQ	HPFHLHGH	LHCHIDWH
Lac 1	IHWHG	WYHSHLSTQ	HPFHLHGH	LHCHIDWH
Lac 3	IHWHG	WYHSHLSTQ	HPLHLHGH	LHCHIDWH
	T 2 T 3	T 3 T 3	T 1 T 2 T 3	T 3 T 3 T 1

Red part indicated the conserved amino acid sequences of copper-binding sites.

Table S2. Information about laccases from *Cerrena unicolor* 6884.

Laccase & Accession number	Gene ID	Count & TPM [1]	Length of cDNA (bp)	Annotation & Accession number	Identity (%)	Integrity
Lac 1 MT720691	TRINITY DN7028 c1 g1	433 11.31725	1326	laccase 1 precursor [Cerrena sp. HYB07] AGK89726.1	86	Lack of the start sequence of 5′
Lac 2 MT720692	TRINITY DN5830 c1 g1	1686.99905 38.687008	1581	laccase 2 precursor [Cerrena sp. HYB07] AID59410.1	79	Yes
Lac 3 MT720693	TRINITY DN1838 c0 g1	711 30.1695	1557	laccase 3 precursor [Cerrena sp. HYB07] AID59411.1	89	Yes
Lac 4 MT720694	TRINITY DN6894 c0 g2	6865.99573848 77.1251736821	1548	laccase 4 precursor [Cerrena sp. HYB07] AID59412.1	94	Yes
Lac 5 MT720695	TRINITY DN6704 c0 g2	197.999694475 10.057269164	1551	laccase [Cerrena unicolor] ALE66000.1	99	Yes
Lac 6 MT720696	TRINITY DN6690 c0 g2	2306.00014 45.060219	1551	laccase [Cerrena sp. WR1] ACZ58369.1	76	Yes

[1] TPM: Transcripts Per Million.

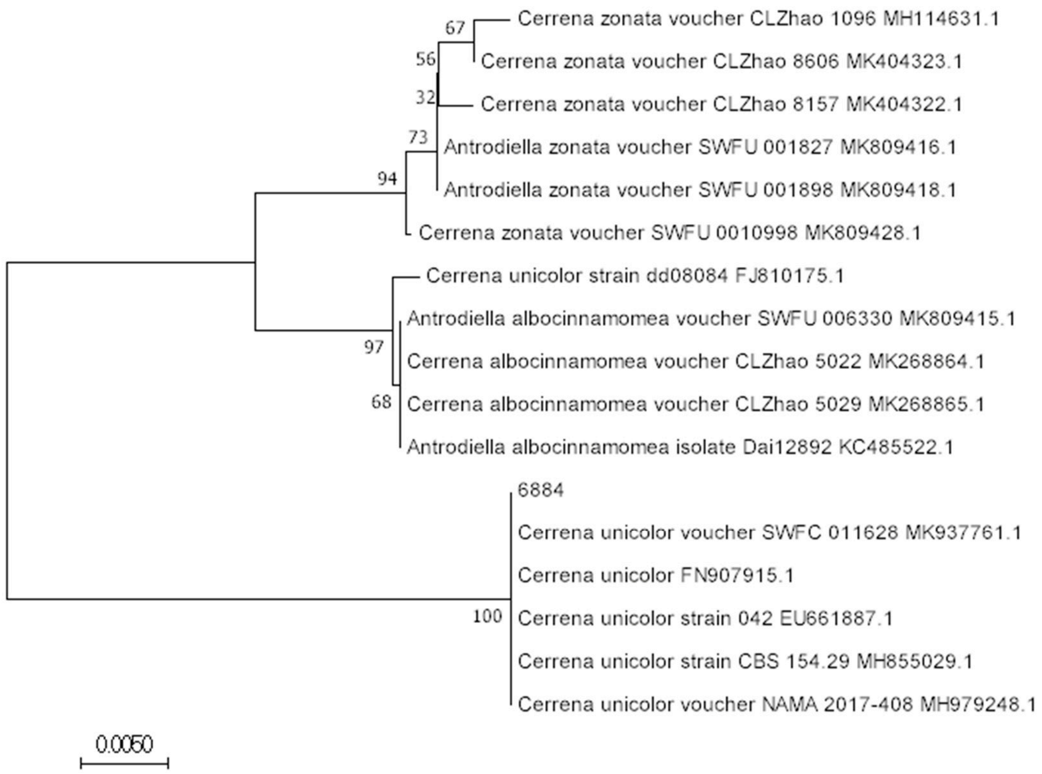

Figure S1. Phylogenetic relationships of *Cerrena unicolor* 6884 and related species based on ITS sequences. The numbers following the strains are accession numbers of ITS sequences. Bootstrap values at nodes are percentages of 1,000 replicates. Scale bar indicates base substitutions/100 bases.

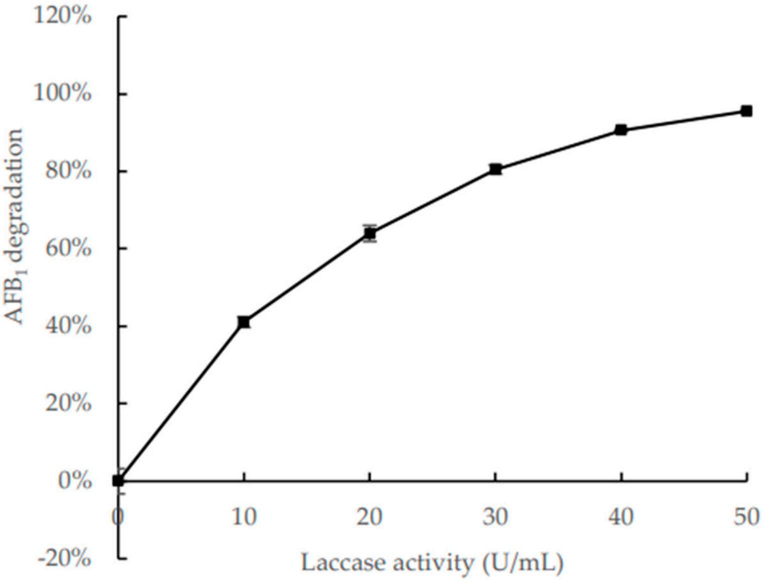

Figure S2. The correclation between Lac 2 from *Cerrena unicolor* 6884 and aflatoxin B1 (AFB_1) degradation. AFB_1 (5 μg/mL) was individually incubated with Lac 2 ranging from 0 U/mL to 50 U/mL in MilliQ water at 45 °C over a period of 60 h. Incubation for 0 h were included as treatment controls. The residual AFB_1 of all samples above was determined by FLD on HPLC (Agilent 1260 Infinity II Series, Agilent Technologies, Waldbronn, Germany). The values represent means ± standard errors (n = 4).

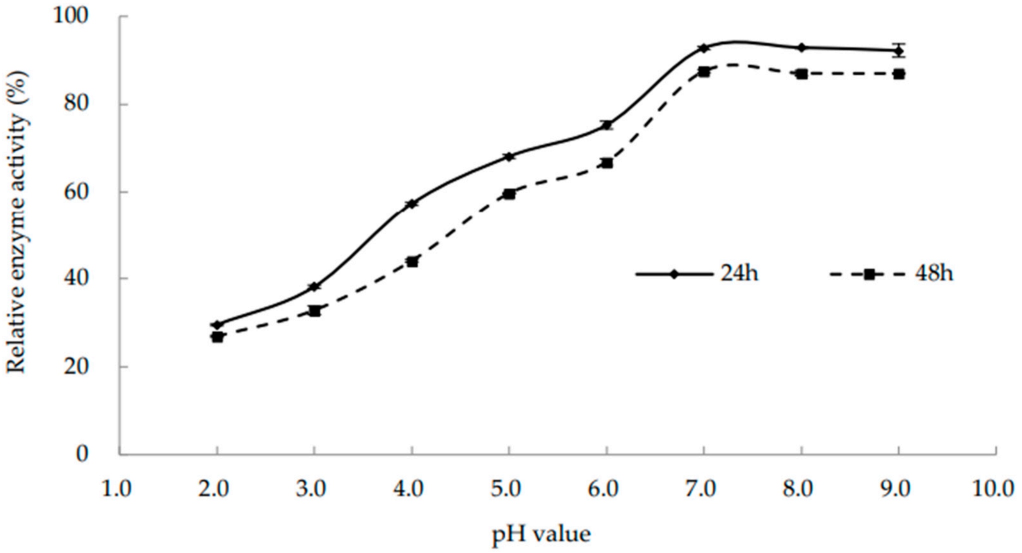

Figure S3. Effects of pH on stability of Lac 2. pH stability was studied by incubating the purified enzyme at different pH values in 20 mM Na2HPO4–citric acid buffers (pH 2.0–7.0), or 20 mM Na2HPO4–NaOH buffers (pH 9.0–10.0) at 25 °C for 24 h or 48 h, and the residual laccase activity was quantified at the optimum pH and temperature using ABTS as the substrate. The activity of the untreated enzyme was taken as 100%. The values represent means ± standard errors (n = 3).

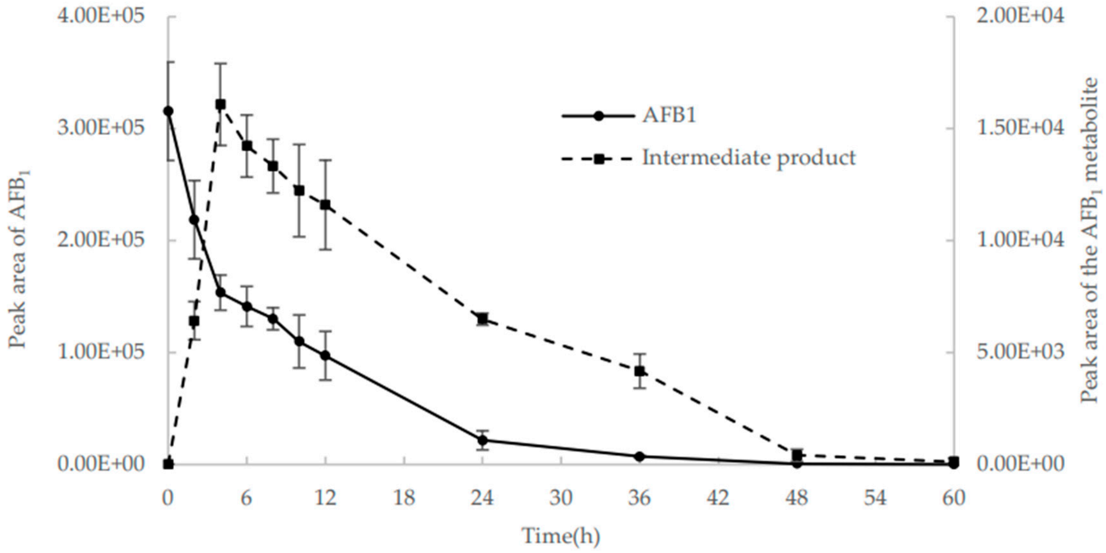

Figure S4. Biological degradation of aflatoxin B_1 (AFB_1) by laccase Lac2 (50U/mL) produced by *Cerrena unicolor* at pH 7.0 and at 45 °C over a period of 0, 2, 4, 6, 8, 10, 12, 24, 36, 48, and 60 h. The residual AFB_1 and intermediate product were determined by UHPLC (Thermo Scientific UltiMate 3000 System, Thermo Fisher Scientific, Germering, Germany). The values represent means ± standard errors (*n* = 3).

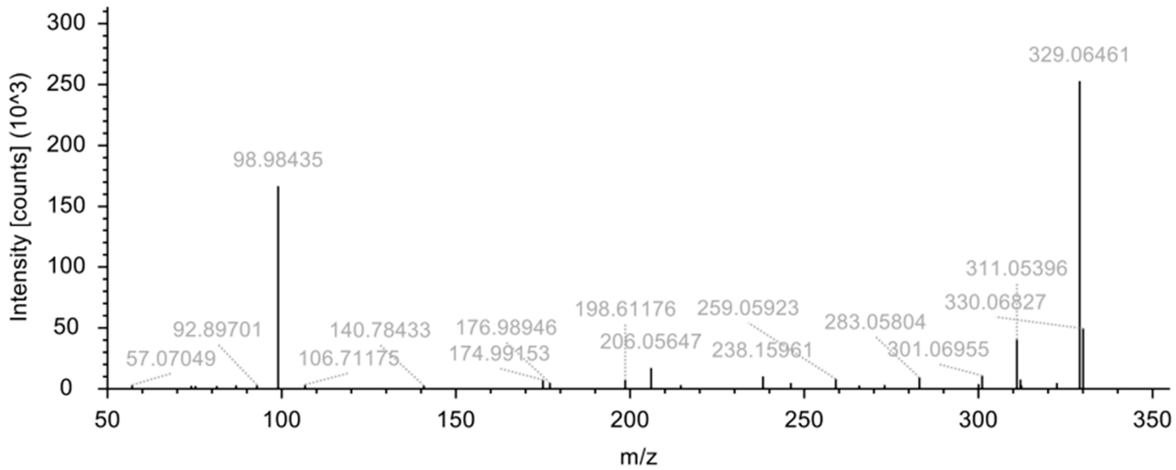

Figure S5. MS/MS spectrum of unknown AFB_1 metabolite.
